# Maternal and Fetal Mechanisms of B Cell Regulation during Pregnancy: Human Chorionic Gonadotropin Stimulates B Cells to Produce IL-10 While Alpha-Fetoprotein Drives Them into Apoptosis

**DOI:** 10.3389/fimmu.2016.00495

**Published:** 2016-12-08

**Authors:** Franziska Fettke, Anne Schumacher, Andrea Canellada, Natalia Toledo, Isabelle Bekeredjian-Ding, Albert Bondt, Manfred Wuhrer, Serban-Dan Costa, Ana Claudia Zenclussen

**Affiliations:** ^1^Department of Experimental Obstetrics and Gynecology, Medical Faculty, Otto-von-Guericke University, Magdeburg, Germany; ^2^University Women’s Clinic, Magdeburg, Germany; ^3^Instituto de Estudios de Inmunidad Humoral, Universidad de Buenos Aires, CONICET-UBA, Buenos Aires, Argentina; ^4^Division of Microbiology, Paul-Ehrlich-Institute, Langen, Germany; ^5^Center for Proteomics and Metabolomics, Leiden University, Leiden, Netherlands

**Keywords:** B cells, pregnancy, hCG, placenta, AFP, hormones, tolerance, IL-10

## Abstract

Maternal immune tolerance toward the fetus is an essential requisite for pregnancy. While T cell functions are well documented, little is known about the participation of B cells. We have previously suggested that IL-10-producing B cells are involved in pregnancy tolerance in mice and humans. By employing murine and human systems, we report now that fetal trophoblasts positively regulate the generation of IL-10-producing B cells. We next studied the participation of hormones produced by the placenta as well as the fetal protein alpha-fetoprotein (AFP) in B cell modulation. Human chorionic gonadotropin (hCG), but not progesterone, estrogen, or a combination of both, was able to promote changes in B cell phenotype and boost their IL-10 production, which was abolished after blocking hCG. The hCG-induced B cell phenotype was not associated with augmented galactosylation, sialylation, or fucosylation of IgG subclasses in their Fc. *In vitro*, hCG induced the synthesis of asymmetrically glycosylated antibodies in their Fab region. Interestingly, AFP had dual effects depending on the concentration. At concentrations corresponding to maternal serum levels, it did not modify the phenotype or IL-10 secretion of B cells. At fetal concentrations, however, AFP was able to drive B cells into apoptosis, which may indicate a protective mechanism to avoid maternal B cells to reach the fetus. Our data suggest that the fetus secrete factors that promote a pregnancy-friendly B cell phenotype, unraveling interesting aspects of B cell function, and modulation by pregnancy hormones and fetal proteins.

## Introduction

B lymphocytes are a major component of the immune system with pleiotropic functions, including antibody production, antigen-presenting capacities, and the secretion of immunomodulatory cytokines. Recent experimental studies of autoimmunity, cancer, and infection diseases have identified B cells with regulatory function, supporting the notion that B cells participate in the maintenance of tolerance to inhibit or downregulate harmful immune responses. This regulatory function is mediated by the production of cytokines, most importantly IL-10, and by the ability of B cells to interact with other cells of the innate and adaptive immune systems (1, 2). Mauri and her colleagues first reported that IL-10-producing B cells suppress Th1 differentiation and prevent autoimmune arthritis development or ameliorate established disease (3). The regulatory effect was dependent upon the release of IL-10 because B cells isolated from IL-10 knockout mice as well as treatment with anti-IL-10 failed to prompt this protective function (4, 5). Carter and colleagues observed that IL-10 B cells established longer contact times with CD4^+^CD25^−^ T cells compared with IL-10 negative B cells and promoted their differentiation into regulatory T cells (Treg) (6). IL-10-producing B cells can also suppress the maturation of dendritic cells (DCs) and influence their cytokine secretion (7).

The concept that IL-10-producing B cells immunomodulate inflammatory processes in autoimmunity brought about the idea that these cells may also control immunological adaptations during mammalian pregnancy. As the fetus is semi-allogeneic to its mother, the maternal immune system has to promote its tolerance while continuing the fight against infection. Failure to accommodate immune adaptations may lead to sporadic or recurrent pregnancy loss. We have recently reported that IL-10-producing B10 cells can restore pregnancy tolerance in a mouse model (8). Accordingly, patients with spontaneous abortion showed a lower proportion of so-called regulatory B cells when compared to normal pregnant patients at the same gestational age (9). *In vitro*, IL-10-producing B cells were able to inhibit the ability of T cells of producing TNF-α (9). Hence, IL-10-producing B cells emerge as regulators of pregnancy immunotolerance. The generation and regulation of these cells has not been explored and emerges as an important issue, yet it can help designing strategies to boost tolerance in patients that suffer from immunological pregnancy losses.

We hypothesize that hormones and other factors secreted by the trophoblast will be relevant in modulating B cell function. Pregnancy-associated hormones, such as the human chorionic gonadotropin (hCG), progesterone (P4), and estradiol (E2), rise dramatically during pregnancy and are essential for successful pregnancy outcome (10). Besides their biological role on preparing the endometrium for implantation [P4; (11)], promoting uterine blood flow and myometrial growth [E2; (12)], and facilitating trophoblast invasion and promoting angiogenesis [hCG; (12–14)], these hormones have been proposed to have immunomodulatory functions (15, 16).

Alpha-fetoprotein (AFP) is produced by the yolk sac and fetal liver (17). Due to its ability to cross the placenta, Alpha-fetoprotein (AFP) is produced by the yolk sac and fetal liver (17). Due to its ability to cross the placenta, AFP levels can be detected not only in the fetus but also within the maternal circulation. AFP is also a tumor marker and is secreted by several carcinomas and has therefore immunosuppressive activities (18). It has not been explored whether AFP contributes to pregnancy success by modulating immune responses.

All of the above support the notion that endocrine factors can modify the immune response and induce fetal tolerance. Here, we devoted to the question whether pregnancy hormones, namely P4, E2, and hCG, as well as the glycoprotein AFP are able of affecting both IL-10 secretion, that would define the modulatory capacity of B cells, and antibody glycosylation patterns that are relevant for defining the quality of humoral response during pregnancy.

## Materials and Methods

### Animals

The use of samples obtained from non-pregnant and pregnant mice was previously approved by the local corresponding authorities (Landesverwaltungsamt Sachsen-Anhalt, Referat Verbraucherschutz, Veterinäramt, Tötungsanzeige). Eight-week-old IL-10eGFP (C57BL/6J background) mice were provided by Dr. Matthias Haury, Instituto Gulbenkian de Ciência, Lisbon upon MTA agreement and further bred in our facilities. Swiss Webster mice were purchased from Charles River (Sulzfeld, Germany) and maintained in our animal facility. C57/BL6 (IL-10eGFP) transgenic females, enabling the identification of IL-10-producing cells by means of green fluorescent protein (GFP) measurement, were allogeneically mated to Swiss Webster males. Mice were checked twice a day for vaginal plug, whose presence indicated day 0 of gestation. Naïve and pregnant mice at day 9 of gestation were sacrificed by cervical dislocation and spleens removed. Day 9 of gestation was chosen to match with the gestation day at which SM-9 cells were generated. Splenic tissue was filtered through a sterile 100 μm cell strainer (BD Falcon, Heidelberg, Germany) and treated with erythrocyte lysis buffer. After lysis, single cell suspension was stained with CD19 PerCp (Biolegend, San Diego, CA, USA) and sorted for CD19 PerCp positive and GFP negative cells by FACS using an FACSAria III (BD, Heidelberg, Germany) while the data were analyzed by DIVA 8.0.1 software.

### Murine B Cell and Trophoblast (SM9-2) Cocultures

The Swiss Webster mouse trophoblastic cell line SM9-2, kindly provided by Joan Hunt and David Wheaton (University of Kansas Medical Center, Kansas City, MO, USA), was cultured in RPMI 1640 medium containing 20% charcoaled fetal bovine serum (FBS), 2 mM l-glutamine, 1 mM sodium pyruvate, 50 μM 2-mercaptoethanol, and 1% penicillin–streptomycin (P/S). Cells were seeded at a concentration of 5 × 10^4^ cells/well on a 24-well culture dish for 24 h (Sarstedt, Newton, NC, USA). Trophoblast attachment to the culture dish was assessed under the microscope, and old medium was carefully removed. To study the effect of trophoblast-derived factor(s) on the generation of IL-10-producing B cells, 500 μl of fresh medium containing 1 × 10^5^ CD19^+^ eGFP^−^ B cells were added to SM9-2 cells at ratio of 2:1 for 24 and 48 h in the presence or absence of mouse CD40 Ligand (R&D Systems, Minneapolis, MN, USA) and CpG ODN M362 (InvivoGen, San Diego, CA, USA). Cell expression of GFP was analyzed by flow cytometry and corresponded to IL-10 production. The 0.5–1 × 10^5^ events were measured in each case.

### Placental Tissue Collection and Processing

Uteri of day 9 pregnant Swiss Webster mice were opened longitudinally and the fetal–placental units were separated from their sites of implantation. Placenta tissue was carefully prepared under a dissecting microscope to remove the decidua basalis and the tissue edges. Each placenta was dissected into four equal sized cubes that were further used in cocultures. The 5 × 10^4^ CD19^+^ IL-10/GFP^−^-sorted B cells were added to placenta explants in 96-well plates with 250 μl of the same media as used for SM9-2 coculture studies. Proliferation of B cells was stimulated by the presence of CpG and CD40L. All cocultures were left for 24 and 48 h. The conversion of IL-10^−^/GFP^−^ cells into IL-10^+^/GFP^+^ B cells was analyzed by flow cytometry. The 0.5–1 × 10^5^ events were measured in each case.

### Human Samples

All experiments involving blood samples from human subjects were previously approved by the Ethics Committee of the Otto-von-Guericke University (study 28/08). Non-pregnant individuals were fully informed about the purpose of this research and provided written consent prior to sampling. Participant characteristics are summarized in Table 1.

**Table 1:** **Participant characteristics.^a^**

Characteristic	Age (mean ± SEM)	Smoking	BMI (kg/m^2^) (mean ± SEM)	Comorbidities (diabetes, hypertension, endocrine disorders)
Participant number				
25	26.4 ± 0.56	1 (6.25%)	21 ± 0.5	0

*^a^Listed are the baseline characteristics for all female, nulliparous participants. Data are presented as means ± SEM*.

### B Cell Isolation and Culture

Peripheral blood mononuclear cells (PBMCs) were isolated from peripheral blood of women who were not pregnant at the moment of blood sampling (Table 1). Using a magnetic separation kit (Miltenyi, Bergisch Gladbach, Germany), B cells were isolated from PBMCs by negative selection. B cell purity was above 95% (Figure S1 in Supplementary Material). B cells (5 × 10^4^ cells/well) were plated in duplicates 24 h before treatment on 96-well plates (Sarstedt, Newton, NC, USA) with 150 μl RPMI 1640 medium supplemented with 3% FBS and 1% P/S. Afterward, cells were either used without stimulation (for hCG treatment and treatment with JEG-3 supernatant) or stimulated with the combination of CD40L (1 μg/ml) and CpG (10 μg/ml) and treated either with physiological concentrations of recombinant hCG (100 mIU/ml, Ovitrelle, Merck Serono, Darmstadt, Germany), P4 (30 ng/ml, Sigma-Aldrich, Taufkirchen, Germany), E2 (1000 pg/ml, Sigma-Aldrich, Taufkirchen, Germany), a combination of P4 and E2, AFP (Abnova, Walnut, CA, USA) at a concentration found in maternal serum (first trimester: 0.015 μg/ml, second trimester: 0.06 μg/ml, and third trimester: 0.2 μg/ml), or AFP at a concentration found in fetal serum (50 μg/ml) for 24 h. Concentrations used were chosen as they represent physiological values during pregnancy, summarized in Table 2. Medium only was used as control. For all experiments cells were maintained at 37°C and 5% CO_2_. A scheme illustrates the culture conditions designed for our paper (Figure S2 in Supplementary Material).

**Table 2:** **Physiological hormone levels and alpha-fetoprotein levels during pregnancy.^a^**

Treatment	hCG 1st trimester	P4 1st trimester	E2 1st trimester	AFP 1st trimester	AFP 2nd trimester	AFP 3rd trimester	AFP fetal
Concentration	5–288,000 mIU/ml	11–90 ng/ml	188–2497 pg/ml	9–18 ng/ml	53–80 ng/ml	98–283 ng/ml	50–3000 μg/ml

*^a^Circulating maternal hormone levels during the first trimester of pregnancy are shown for human chorionic gonadotropin (hCG), progesterone (P4), and estradiol (E2). The serum concentration of fetal alpha-1-fetoprotein (AFP) and maternal serum concentrations for AFP are listed for all the three trimesters of pregnancy*.

### Cell Staining and Flow Cytometry

Harvested human cells were stained for the extracellular markers CD19 (FITC), CD27 (PerCp), and CD24 (AF647) (Biolegend, San Diego, CA, USA). Following O/N fixation, permeabilization (eBioscience, San Diego, CA, USA), and intracellular IL-10 (PE) (Biolegend, San Diego, CA, USA) staining, cells were analyzed by flow cytometry. A total of 1 × 10^4^ events were measured in each case. CD27^+^ and CD24^high^ cells were gated, and within this population, the coexpression of CD19 and IL-10 was recorded. Samples were measured using a FACS Calibur (BD, Heidelberg, Germany), while the data were analyzed by BD CellQuest Pro software.

### Cytokine and Immunoglobulin Enzyme-Linked Immunosorbent Assay

Supernatants from B cells were collected after 24 h (cytokines) or 12 days (immunoglobulin; Ig) (Figure S2 in Supplementary Material). IL-10 cytokine secretion was determined using a human IL-10 OptEIA™ Enzyme-Linked Immunosorbent Assay (ELISA) kit (BD Biosciences, Heidelberg, Germany). Human IgM, IgA, and IgG levels were quantified using Ig ELISA kits from Bethyl Laboratories (Montgomery, TX, USA). All steps were performed according to the technical data sheet/manufacturer’s protocol. Cytokine and Ig levels for duplicate samples were quantified and analyzed using a BioTek Synergy HT Multi-Mode Microplate Reader with Gen5 software.

### Apoptosis/Cell Viability Assay

Harvested B cells were washed with cold PBS and stained with FITC Annexin V and propidium iodid as indicated by manufacturer’s instructions (BD Pharmingen™ FITC Annexin V Apoptosis Detection Kit I, Heidelberg Germany). Data were analyzed by flow cytometry within 1 h.

### Caspase Activity Assay

We measured caspase-3 and -7 activities using a Caspase-Glo 3/7^®^ assay (G8091, Promega, Madison, WI, USA) following treatment of B cells with AFP. Cell culture plates containing cells were harvested and set aside to equilibrate to RT for 30 min. The 100 μl of Caspase-Glo^®^3/7 reagent had been added to each well, the contents of wells were gently mixed using a plate shaker at 300–500 rpm for 30 s. Following incubation at RT for 1 and 3 h, the luminescence was measured using a BioTek Synergy HT Multi-Mode Microplate Reader according to the manufacturer’s instructions.

### Human B Cell and Trophoblast (JEG-3) Cocultures

The hCG-producing human choriocarcinoma trophoblast cell line (JEG-3) purchased from Cell Line Service (CLS, Eppelheim, Germany) was cultured in RPMI 1640 medium supplemented with 10% FBS and 1% P/S. Cells were trypsinized and seeded at a concentration of 5 × 10^4^ cells/well on a 24-well culture dish for 24 h (Sarstedt, Nümbrecht, Germany). Human B cells also incubated for 24 h were harvested, suspended in fresh charcoaled medium, and cocultured (1:1) with JEG-3 cells in the presence or absence of 1 μg/ml CD40L and 10 μg/ml CpG for further 24 h. To study the influence of hCG on the expansion of pregnancy protective IL-10-producing B cells, an anti-hCG antibody (Santa Cruz, San Francisco, CA, USA), blocking hCG from binding to its target receptor, was added to cell cultures. Cells were stained for CD19, CD24, CD27, and IL-10 as indicated previously and analyzed by flow cytometry. Supernatants were collected for analysis of IL-10 by ELISA.

### IgG Glycosylation Analysis

The glycosylation of the IgGs from the culture were analyzed as described previously with minor modifications (19). Briefly, human IgGs were affinity captured from the culture medium using CaptureSelect IgG-Fc (Hu) beads (Invitrogen, Bleiswijk, The Netherlands). After elution with 100 mM formic acid (Fluka, Steinheim, Germany), samples were dried by vacuum centrifugation. The purified antibodies were sequentially reconstituted in 20 μl 50 mM ammonium bicarbonate buffer (pH 8.0; Sigma-Aldrich, Taufkirchen, Germany) in 15% acetonitrile (Biosolve BV, Valkenswaard, The Netherlands). After 5 min of incubation on a multiwell plate shaker, 20 μl ultrapure water containing 330 ng sequencing grade modified trypsin (Promega, Madison, WI, USA) was added, followed by a further 5 min of incubation on a shaker and O/N incubation at 37°C. The resulting tryptic digest was subjected to subclass specific glycopeptides analysis by LC-MS. Analysis was performed with nanoLC-reversed phase (RP)-electrospray (ESI)-ion trap (IT)-MS(/MS) on an Ultimate 3000 RSLCnano system (Thermo Scientific, Dreieich, Germany) coupled with an amaZon speed ESI-IT-MS (Bruker Daltonics, Bremen, Germany). A precolumn (Acclaim PepMap 100 column, 100 μm × 20 mm, particle size 5 μm, Thermo Scientific) was used to wash and concentrate the sample, and separation was achieved on an Acclaim PepMap RSLC C18 nanocolumn (75 μm × 150 mm, particle size 2 μm, Thermo Scientific, Dreieich, Germany) with a flow rate of 700 nl/min. The following linear gradient was used, with solvent A consisting of 0.1% formic acid in water and solvent B of 95% acetonitrile, 5% water: *t* = 0 min, 3% solvent B; *t* = 5 min, 1% B; *t* = 20 min, 27% B; *t* = 21 min, 70% B; *t* = 23 min, 70% B; *t* = 24 min, 3% B; and *t* = 43 min, 3% B. The sample was ionized in positive ion mode with a Captive sprayer (1200 V) with a tapered tip (20 μm ID). The solvent was evaporated at 180°C with a nitrogen flow of 3 l/min. A nanoBooster (Bruker Daltonics) was mounted onto the source and saturated the nitrogen flow with ACN to enhance the sensitivity (0.2 bar). The MS1 ion detection window was set at 550–1800 *m*/*z*. IgG Fc-glycopeptides were identified based on their retention times and the monoisotopic masses of the protonated species. Internal calibration of LC-MS spectra was performed in Bruker Data Analysis 4.0 using a list of known glycopeptides, before exportation of the data to the mzXML format, extraction, and exploration in Microsoft Excel.

### Hybridoma Cell Culture

Anti-DNP MAb-secreting hybridomas were prepared and characterized as originally described by Köhler and Milstein and mentioned in the study by Margni and colleagues (20). Fusion partners were spleen cells from BALB/c mice immunized with dinitrophenylated human gamma globulin (DNP-HGG) and the NSO myeloma cell line. A hybridoma secreting symmetrically and asymmetrically glycosylated anti-DNP antibodies of the IgG1 subclass was selected (21). Cells were suspended in RPMI 1640 medium (supplemented with 10% FBS and NaHCO_3_ 2 g/l, 20 U/ml penicillin, and 20 mg/ml streptomycin) at 5 × 10^5^ cells/ml and incubated in the presence or absence of recombinant hCG (50 mIU/ml, Ovitrelle). Cultures were performed as triplicates in 96-well flat-bottom plates (Corning Inc., Corning, NY, USA). After 24 or 48 h of incubation, the cell culture supernatants were harvested and processed as described below.

### Assessment of Asymmetric Fab Glycosylation of Anti-DNP Antibody by Con A-Binding Assay

The property of the carbohydrate prosthetic group present in the Fab region of asymmetrically glycosylated IgG molecules to bind steadily to Concanavalin A (Con A) was applied taking into account that this lectin binds molecules containing α-d-mannopiranosyl, α-d-glucopiranosyl, and sterically related residues (22). Samples were diluted 1:10 in Con A buffer (0.025 M Tris–HCl, 0.2 M NaCl, and 0.003 M each of CaCl_2_, MgCl_2_, and MnCl_2_; 0.02% Na_3_N; pH 7.2), mixed with appropriate volume of either Con A buffer, or Con A-Sepharose suspension (Sigma, 1 ml of 50% gel suspension/mg of protein) and kept for 2 h at 4°C with occasional agitation. After supernatant collection, total as well as unbound anti-DNP IgG was measured by ELISA as described previously (23). The percentage of asymmetrically glycosylated IgG1 monoclonal antibody (mAb) in the hybridoma culture supernatants was calculated from the statistical linear regression analysis of both ELISA line draws.

### Statistical Analysis

Data were examined for normality using the Kolmogorov–Smirnov test; results are expressed as the means or means ± SEM. Differences within the groups were compared using repeated measures one-way ANOVA. Where multiple comparisons were made, Bonferroni adjustment was applied/Bonferroni multiple post *t* test were applied to evaluate the differences of means of multiple groups. Statistical testing was carried out using Prism 5 software (GraphPad Software, 2007 edition, La Jolla, CA, USA). A confidence interval of 95% or a *p*-value of <0.05 was considered significant and indicated as *, *p* < 0.01 indicated as **, and *p* < 0.001 indicated as ***.

## Results

### Trophoblasts Themselves or Trophoblast-Derived Factors Promoted the Generation of Murine IL-10-Producing B Cells *In Vitro*

**Figure 1:**
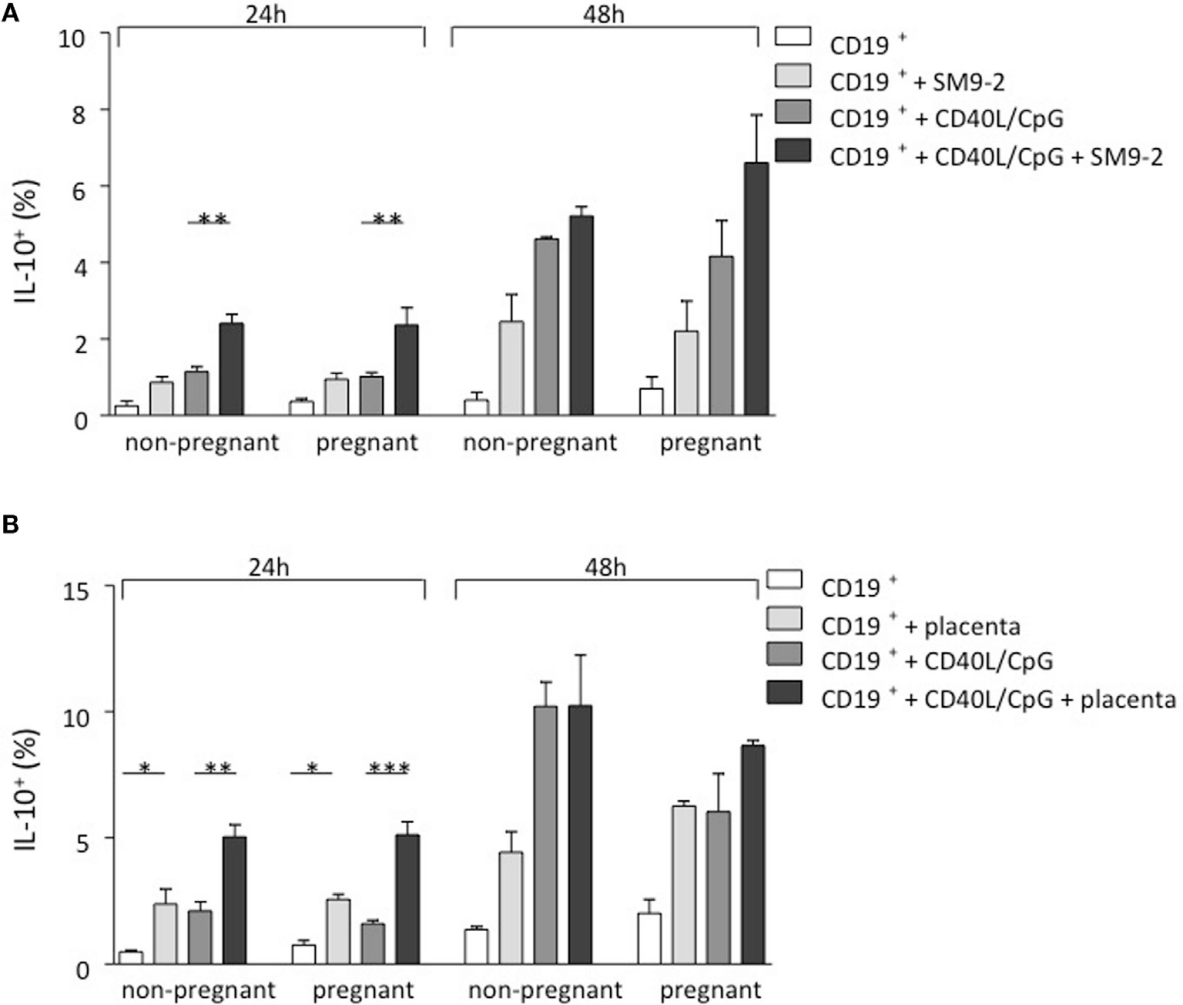
**Trophoblasts themselves or trophoblast-derived factors promoted the generation of murine IL-10-producing B cells *in vitro***. **(A)** Naive and pregnant (day 9) female IL-10eGFP (C57BL/6 background) mice were sacrificed and total CD19^+^ GFP^−^ B cells isolated. CD40L/CpG-stimulated B cells as well as unstimulated controls were cocultured for 24 h with murine trophoblast cells (SM9-2). The generation of IL-10-producing B cells was calculated by evaluating IL-10 expression in total B cells using flow cytometry. After coculture of B cells with trophoblast cells, a significant conversion of IL-10^−^ into IL-10^+^ B cells compared to stimulated cells cultured alone was observed. Overall, the number of IL-10^+^ B cells increased with incubation time. Comparing the frequency of generated IL-10^+^ B cells within both groups demonstrated no statistical significance, whether CD19^+^eGFP^−^ B cells were obtained from naïve or pregnant mice following 24 and 48 h culture. **(B)** Here, isolated CD19^+^ GFP^−^ B cells were cocultured with placenta explants from pregnant day 9 Swiss Webster mice for 24 and 48 h. The generation of IL-10-producing B cells was calculated by evaluating IL-10 expression in total B cells using flow cytometry. The 24 h coculture of CD19^+^eGFP^−^ B cells with placenta explants demonstrated a significant conversion into IL-10^+^ B cells. This effect was observed, whether B cells has been stimulated or not and the overall number of IL-10^+^ B cells increased with incubation time. Comparing the frequency of generated IL-10^+^ B cells within each group demonstrated no statistical significance, whether CD19^+^GFP^−^ B cells were obtained from naïve or pregnant mice following 24 and 48 h culture **(B)**. Data are presented as means + SEM. Statistical analysis was carried out by repeated measures two-way ANOVA followed by Bonferroni correction for multiple analysis (*p ≤ 0.05, **p ≤ 0.01, and ***p ≤ 0.001).

As IL-10-producing B cells were recently proposed to be important regulators of feto-maternal tolerance and knowing that the fetus itself, and most specifically, the trophoblast, secretes modulating factors, we first studied whether trophoblasts were able to influence B cell phenotype and intracellular IL-10 production in a mouse model. For this, we isolated splenic CD19^+^GFP^−^ B cells from transgenic modified C57BL/6 (IL-10eGFP) mice whose IL-10 secreting cells can be visualized as they express GFP. We employed either virgin mice or pregnant mice (day 9 of pregnancy). Unstimulated B cells as well as CpG/CD40L-stimulated B cells were cocultured with the murine trophoblast cell line SM9-2 at a ratio of 2:1 for 24 and 48 h. This cell line was originally generated from placentas of pregnant mice at day 9 of gestation. The conversion of IL-10^−^ cells into IL-10^+^ cells was analyzed and quantified by flow cytometric detection of GFP. We observed a relevant conversion of IL-10^−^ B cells into IL-10^+^ B cells after coculture of trophoblasts with B cells (Figure 1A). The generation of IL-10-producing B cells from total cells was observed in both B cells obtained from virgin or pregnant cell donors (Figure 1A). This confirms the important role of trophoblast cells and or their factors in converting B cells into B cells with the ability of produce IL-10.

In order to replicate a more physiological setting, the second experimental design utilized placental tissue derived from pregnant Swiss Webster mice on day 9 instead of a trophoblast cell line. Placenta explants were obtained from pregnant day 9 Swiss Webster mice. CD19^+^GFP^−^ B cells were collected from virgin or 9 day pregnant C57BL/6 (IL-10eGFP) mice previously mated with Swiss Webster male and incubated with placenta explants for 24 or 48 h. As shown in Figure 1B, the presence of placenta explants induced the differentiation of IL-10 negative B cells into B cells able to secrete IL-10. This effect was observed independently of whether the cell donors were pregnant or not (Figure 1B). Hence, fetal trophoblasts generate factors that are able to promote the *de novo* generation of IL-10-producing B cells that are important for fetal tolerance.

### Trophoblast and Trophoblast-Derived Soluble Factors Promoted the Generation of Human IL-10-Producing B Cells *In Vitro*; This Effect Was Abrogated by Blocking hCG

**Figure 2:**
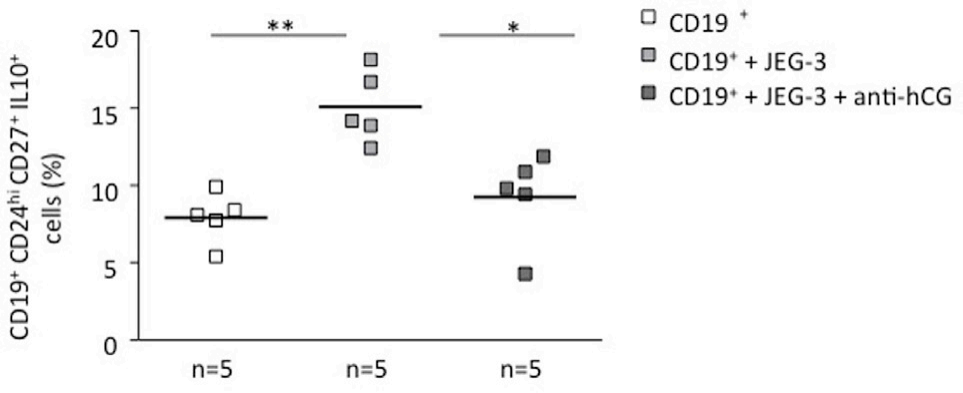
**Trophoblasts or trophoblast-derived factors promoted the generation of human IL-10-producing B cells *in vitro*: this effect could be abrogated by blocking hCG**. CD19^+^ isolated B cells from PBMCs obtained from non-pregnant female donors were cultured in the presence of JEG-3 trophoblast cells. A significant increase in the proportion of CD19^+^CD24^hi^CD27^+^IL-10^+^ cells was registered by flow cytometry. The blockage of hCG by using an anti-hCG antibody abrogated this effect. Each square represents one single subject. Data are presented as single values/donor and means. Statistical analysis was carried out by repeated measures one-way ANOVA followed by Bonferroni correction for multiple analysis (*p ≤ 0.05 and **p ≤ 0.01).

To understand whether the phenomenon observed in a murine system is relevant for human pregnancies, we next we cocultured stimulated total B cells with human choriocarcinoma trophoblastic cells (JEG-3) for 24 h. Upon flow cytometry evaluation, we observed a statistically significant increase in the number of CD19^+^CD24^hi^CD27^+^IL-10^+^ cells (Figure 2) and active secretion of IL-10 into the supernatant (Figure S3 in Supplementary Material). JEG-3 supernatant was also able to induce IL-10 secretion by isolated CD19^+^ cells (Figure S4 in Supplementary Material), which suggests a soluble factor as responsible for this. As we recently showed that hCG secreted by JEG-3 has an important effect in the number and functionality of T cells (15), we next aimed to investigate whether hCG was the mediator of the effect in B cell phenotype. For this, we blocked hCG using an anti-hCG antibody, which indeed abrogated JEG-3-induced increase of IL-10-producing B cells within total B cells (Figure 2). Thus, trophoblast-derived hCG is able to change the phenotype of B cells and turn them pregnancy protective.

### hCG, but Not P4 or E2, Could Induce a B Cell Phenotype Change and an Increase in the Number of IL-10-Producing B Cells

**Figure 3:**
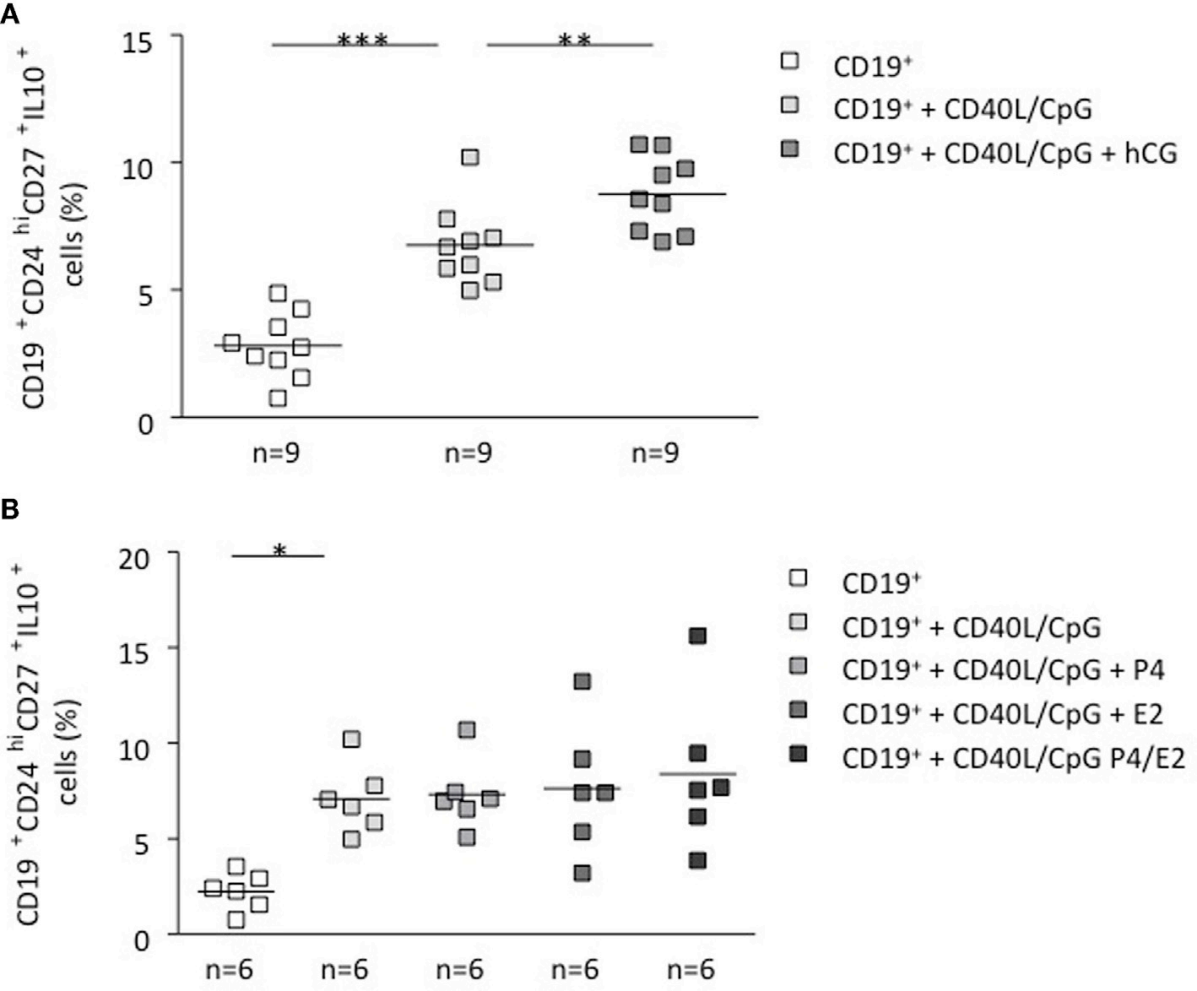
**hCG induced a B cell phenotype change and an increase in the number of IL-10-producing B cells**. **(A)** Following magnetic isolation of untouched CD19^+^ B cells from human PBMCs, B-lymphocytes were cultured for 24 h in charcoaled medium (control) or charcoaled medium with stimulation (CD40L/CpG) in the presence of recombinant hCG (100 mIU/ml), P4 (30 ng/ml), E2 (1000 pg/ml), or the combination of P4 and E2. Extracellular B cell markers as well as intracellular IL-10 production were evaluated by flow cytometry. *In vitro* application of recombinant hCG (100 mIU/ml) to total B cells provoked a significant increase in the CD19^+^CD24^hi^CD27^+^IL-10^+^ cell population. **(B)** Incubation of B cells with P4, E2, or a combination of both did not alter their phenotype. Each square represents one single subject and means are showed. Statistical analysis was carried out by repeated measures one-way ANOVA followed by Bonferroni correction for multiple analysis (*p ≤ 0.05, **p ≤ 0.01, and ***p ≤ 0.001).

To unequivocally confirm the participation of hCG in the induction of a pregnancy protective phenotype, we stimulated total B cells with CD40L (5 μg/ml) and CpG (10 μg/ml), which were further cocultured *in vitro* with recombinant hCG. To evaluate whether other pregnancy-relevant hormones are also able to influence the phenotype of B cells, we repeated the experiments and incubated total B cells with P4, E2, or a combination of the latter for 24 h. We observed a significant increase in the CD19^+^CD24^high^CD27^+^IL-10^+^ cell population when adding recombinant hCG compared with cells cultured without hormones (Figure 3A). Interestingly, and as we reported before (9), it seems that hCG has an additive effect to CD40L/CpG as treatment with both boosts IL-10 production in a magnitude that is higher than the addition of any of them alone (Figure S4 in Supplementary Material). The addition of P4, E2 had no statistically relevant effect in generating this cell population out of total B cells (Figure 3B). Thus, similarly as trophoblasts or trophoblast supernatant, recombinant hCG, but not P4 and E2, are able to generate B cells that express extracellular markers previously related to so-called regulatory B cells. Additionally, these cells actively secrete IL-10 as we could observe using flow cytometry (Figure S5 in Supplementary Material).

### Neither hCG Nor Other Pregnancy Hormones Were Able to Affect the Concentration of Igs

We next aimed to understand whether the effect of hCG was exclusively related to IL-10-producing B cells involved in cellular immunology or whether hCG was able to influence the Ig production by B cells cultured for 12 days, so that they produce antibodies. As tested *in vitro*, the addition of exogenous pregnancy hormones and AFP had no impact on Igs, as we found no differences in the levels of human IgM, IgA, and IgG in supernatants from isolated B cells cultured for 12 days with or without the addition of hCG, P4, E2, P4 plus E2, or AFP at different concentrations (Figure S6 in Supplementary Material).

### The Fc Glycosylation Profile of IgG Subclasses Was Unaffected by Pregnancy Hormones and AFP

After observing that hCG, but none of the other tested hormones, was able to influence the phenotype of B cells albeit not their ability to secrete cytokines, we next intended to clarify whether hCG is able to influence the glycosylation profile of IgG, thereby affecting the quality of the immune response. For this, we isolated human serum IgG in order to assess the subclass-specific glycosylation profiles and potential differences based on the addition of pregnancy hormones. The glycosylation profile of the Fc peptides featured galactosylation, sialylation, and fucosylation for IgG1 and for IgG2 and IgG3 combined. The analysis revealed no differences in the *N*-glycosylation after treatment with recombinant pregnancy hormones or different AFP concentrations and subsequent 12-day culture (Figures S7A,B in Supplementary Material; glycosylation profiles for combined IgG2/3 are not shown). This indicates that Fc glycosylation processes are not affected by pregnancy hormones and AFP *in vitro*.

### hCG Increased Fab Asymmetric Glycosylation of Hybridoma 112D5 Cells

**Figure 4:**
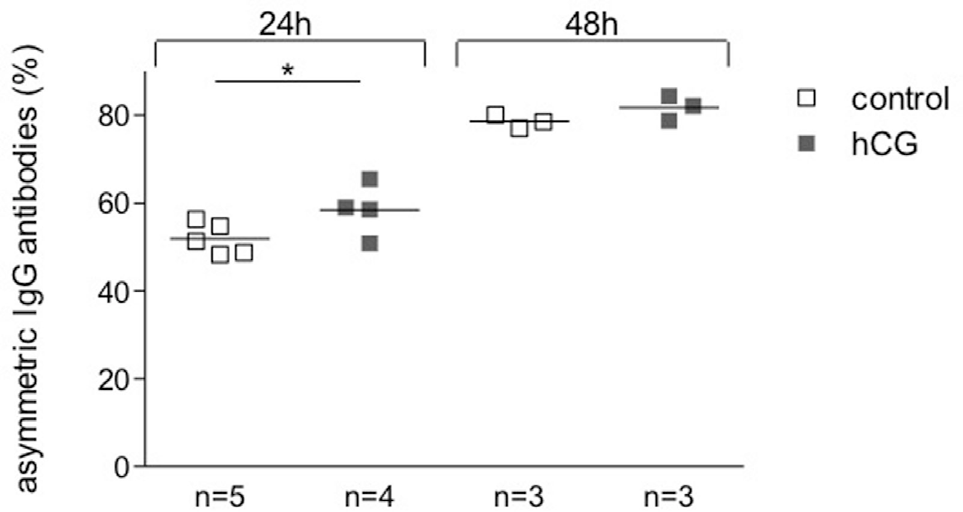
**The addition of hCG augmented the proportion of AAbs**. A hybridoma secreting symmetrically and asymmetrically glycosylated anti-DNP antibodies of the IgG1 subclass was selected for this experiment. Cells were incubated in the presence or absence of recombinant hCG (Ovitrelle, 50 mIU/ml). After 24 and 48 h of incubation, the supernatants were harvested and processed. Percentages of total and symmetric IgG1 monoclonal antibodies in the hybridoma culture supernatants were assessed by Con A-binding assay followed by ELISA. The proportion of AAbs was calculated based on the difference between total and symmetric antibodies and depicted as percentage. Results are shown as single values with means from three experiments. Statistical analysis was carried out by two-way ANOVA followed by Bonferroni corrections for multiple comparisons (*p ≤ 0.05).

To determine a potential effect of hCG on the synthesis of pregnancy protective AAbs, we sampled supernatant from hybridoma 112D5 cells following culture for 24 and 48 h with recombinant hCG at the same concentrations that were effective in influencing B cell phenotype or standard serum as controls. We were able to show that the addition of recombinant hCG provoked an enhancement in the percentage of IgG1 anti-DNP antibodies synthesized 112D5 by hybridoma cells that were able to steadily bound to Con A following 24 h culture (Figure 4). These results confirm the ability of hCG to induce the synthesis of AAbs, namely IgG antibodies asymmetrically glycosylated in their F(ab).

### AFP at Maternal Concentrations Had No Effect on B Cell Phenotype or IL-10 Production, but Fetal Concentrations of AFP Drove B Cells into Apoptosis

**Figure 5:**
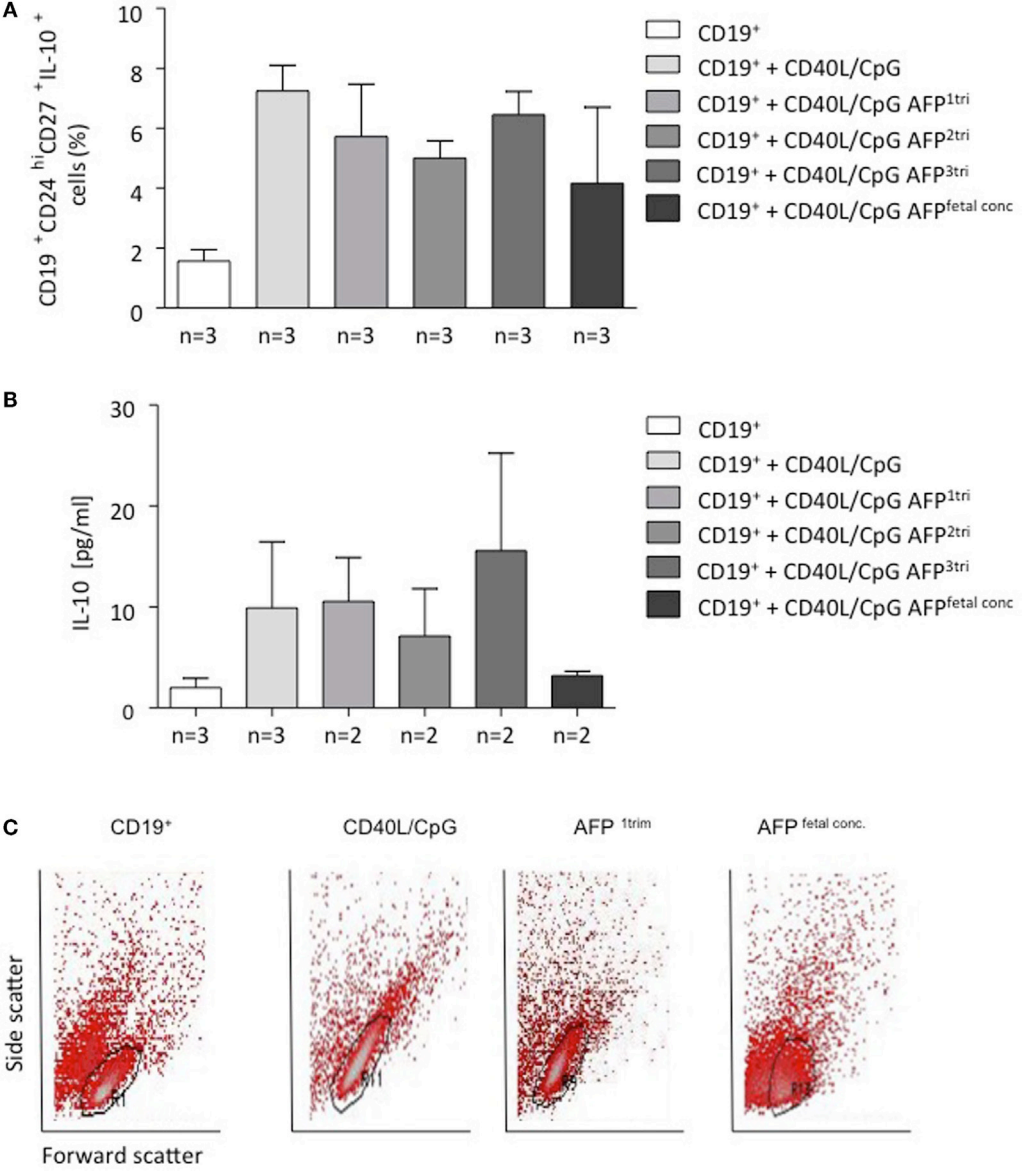
**AFP at maternal or fetal concentrations did not affect the proportion of CD19^+^CD24^hi^CD27^+^IL-10^+^ cells within B cells, while AFP at fetal concentrations caused a positional shift within the total B cell population**. **(A)** CD19^+^CD24^hi^CD27^+^IL-10^+^ cell numbers within isolated B cells remained unchanged following application of AFP at maternal or fetal serum concentration. **(B)** The addition of AFP had no impact on the IL-10 secretion investigated in the culture supernatants by ELISA. **(C)** We did observe a change in the actual dot plot analysis following addition of fetal serum AFP levels. CD24^hi^ and CD27^+^ cells were identified on an FSc vs. SSc dot plot using free-drawn regions. The population could be easily identified following application of standard medium, CD40L/CpG-stimulated medium, and the combination of CD40L/CpG and maternal serum AFP level during first trimester. However, it was not possible to identify this double-positive population following the addition of fetal serum AFP levels. Data are either presented as means plus SEM **(A,B)** or FSc vs. SSc dot plot using a free-drawn region. Statistical analysis was carried out by repeated measures one-way ANOVA followed by Bonferroni correction for multiple analysis. Flow cytometry data were analyzed using BD CellQuest Pro software.

**Figure 6:**
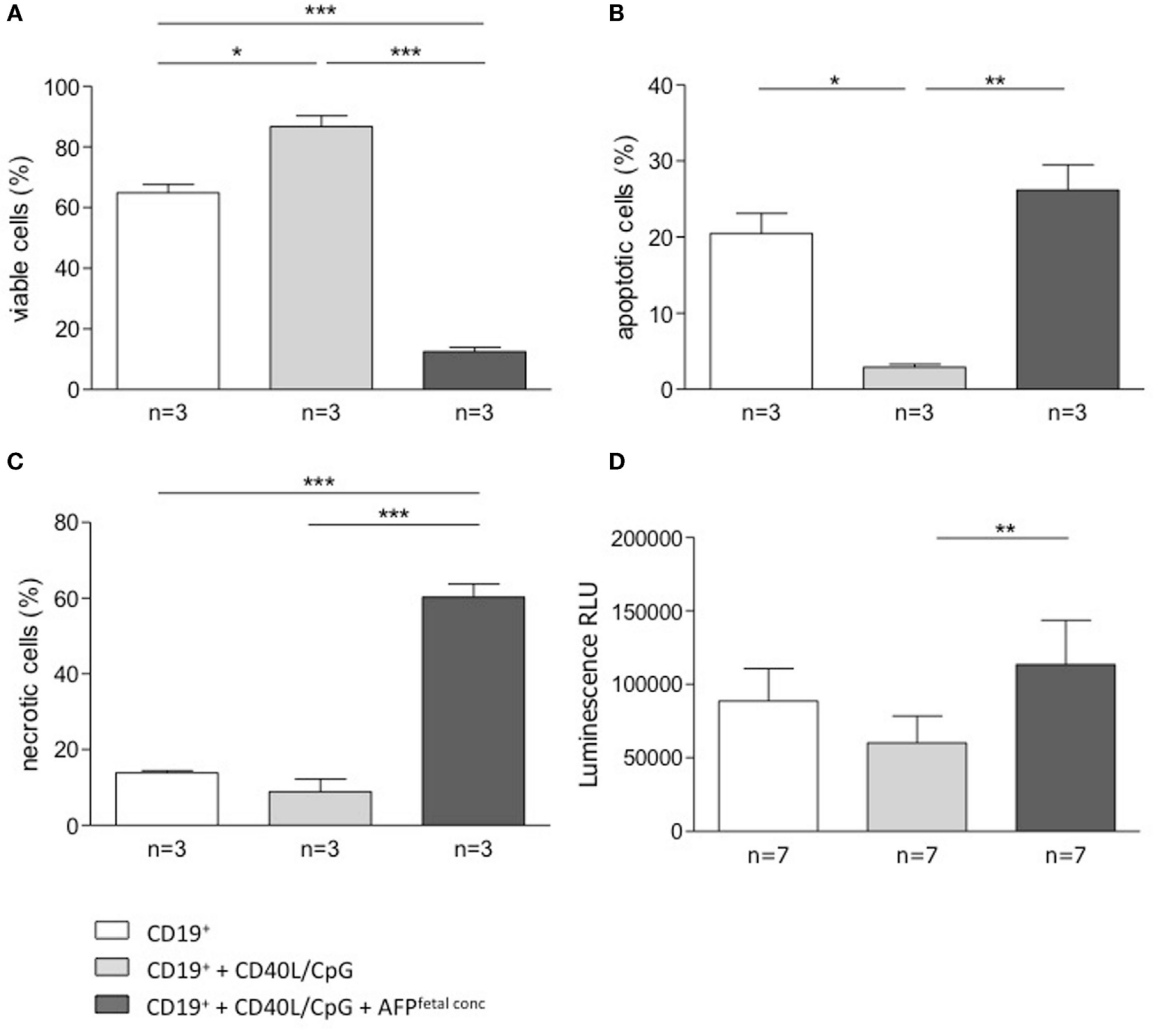
**AFP added at fetal concentration induced apoptosis and necrosis in total B cells**. **(A–C)** 5 × 10^4^ isolated B cells were stimulated by CD40L/CpG (gray bars), stimulated and exposed to 50 μg/ml AFP (black bars), or incubated in standard medium as a control (white bars) for 24 h. The percentage of viable, apoptotic, and necrotic cells in the total B cell population was assessed using the FITC Annexin V Apoptosis Detection kit with flow cytometric analysis. Assessment of B cell membrane asymmetry following AFP application demonstrated a significant decrease in the percentage of viable cells and a significant increase in the percentage of apoptotic and necrotic cells compared to the application of stimulated medium. **(D)** Measurements of caspase activity using the Caspase-Glo^®^ 3/7 assay showed a significant increase in caspase-3 and -7 activities following AFP treatment compared with CpG/CD40L treatment only. Results are expressed as a percentage or Luminescence RLU and presented as means ± SEM. Statistical analysis was carried out by repeated measures one-way ANOVA followed by Bonferroni correction for multiple analysis (*p ≤ 0.05, **p ≤ 0.01, and ***p ≤ 0.001).

To understand whether AFP, which is relevant during embryonic life, is also important for B cell function, we tested whether AFP influences B cell phenotype and IL-10 production. For this, we added recombinant AFP in concentrations corresponding to the physiological values observed in serum for the three trimesters as well as to the fetal concentrations to total B lymphocytes from peripheral blood of non-pregnant women*. In vitro* stimulation of total B cells with AFP at maternal or fetal concentrations had no significant effect on the cellular phenotype of B cells or their IL-10 secretion (Figures 5A,B). However, when examining the dot plots, we observed a shift in the cell population induced by the fetal concentration of AFP that suggested cell death (Figure 5C). To deeper investigate this interesting observation, B cells were treated with fetal concentrations of AFP and after 24 h, stained with an Annexin V and PI. As shown in Figure 6, treatment with 50 μg/ml AFP-induced apoptosis (Figures 6A,B) and cell death (Figure 6C) of cultured total B cells to a great extent, whereas significantly more viable cells remained following culture in standard medium alone or with the addition of CD40L/CpG (Figure 6A). To assess whether caspase activity is influenced by AFP, we employed a Caspase-Glo^®^ 3/7 luminescent assay. As shown in Figure 6D, AFP at fetal concentrations augmented caspase-3 and -7 activity of B cells, while supplementation with CpG and CD40L inhibited these enzymes as anticipated (see also Figure S8 in Supplementary Material). Hence, AFP has no effect on B cells when tested at maternal concentrations. Used at fetal concentrations, however, it drives B cells into apoptosis. We speculate that this mechanism may provide a protective barrier for maternal B cells that try to reach the fetus.

## Discussion

The quality of the maternal immune response during pregnancy dictates its outcome. Important players are cells of the innate and adaptive immune system and the cytokines they produce. B cells and in particular B cells with the ability to produce IL-10 emerge as novel mediators of pregnancy tolerance (24, 25) and understanding the pathways that lead to their generation is vital to evaluate their possible therapeutic application.

Here, we hypothesized that molecules produced by the fetal trophoblast are able to regulate the phenotype of B cells and their ability to produce the immunomodulatory molecule IL-10. We focused our attention particularly on pregnancy hormones as B cells express the respective hormone receptors. Very interestingly, our results confirm that from all hormones, hCG is the only one able to convert CD19^+^ cells into IL-10-producing B cells and to also affect the capability of plasma cells in secreting AAbs. Since from all tested hormones, hCG is unique in its property to be exclusively secreted by the placenta during pregnancy, our data confirm the active participation of the fetus in establishing and maintaining its own tolerance. B cells are not only pregnancy protective but can also synthesize antibodies that, if reaching the fetus, can danger pregnancy outcome or fetal health (26, 27). We found that fetal concentrations of AFP drive B cells into apoptosis. This suggests the existence of fetal mechanisms that regulate the entrance of putatively dangerous B cells and so avoid their negative effects. Overall, our data show that the fetus is able to modulate the maternal immune response to its own benefit.

We first undertook studies using a murine system. In particular, we took advantage of a mouse strain that allowed us to negatively select and exclude GFP^+^/IL-10^+^ B cells. If the cultured non-producing IL-10 cells began secreting it, their detection was possible by measuring GFP in the system. This way we could prove a significant conversion of IL-10 negative into IL-10-producing B cells after *in vitro* coculture with an allogeneic trophoblast cell line. The same result could be obtained when using semi-allogeneic placenta explants. Hence, trophoblast-secreted factors can stimulate the IL-10 production by B cells. The anti-inflammatory cytokine IL-10 has a wide range of functions. Principally, it inhibits the production of pro-inflammatory cytokines, thus shifting the seesaw toward a Th2 response by inhibiting the production of Th1 cytokines. In the 1980s and 1990s, much attention was paid to the cytokine ratio and a central role for Th2 vs. Th2 cytokine was proposed for pregnancy to be successful (28). With the first papers reporting dispensability of Th2 cytokines for murine pregnancy (29) this concept changed and the attention was rather focused on cellular mechanisms leading to active tolerance to the fetal antigens (30, 31) rather than cytokine balance. Nevertheless, even though the absence of IL-10 itself does not jeopardize tolerance establishment (29), IL-10 seems to be relevant to reverse the harmful effect of lipopolysaccharide (LPS)-driven pregnancy loss (32, 33). In IL-10-deficient mice, the pro-inflammatory cytokine TNF-α is mediating the adverse effects of LPS in pregnancy (34). Additionally, fetuses born from IL-10-deficient mothers were growth restricted (32), so that it can be concluded that while IL-10 may not be essential to pregnancy to come to end, it is needed for proper fetal growth and to protect from infections.

Using cells from non-pregnant female donors, we could confirm that trophoblast-secreted factors were able to augment the proportion of so-called regulatory B cells, namely, IL-10-producing CD19^+^CD24^hi^CD27^+^ cells. We next sought to understand which trophoblast-secreted mediators are involved in the stimulation of IL-10 production by B cells. Having recently reported that the hormone hCG, produced by trophoblasts, is able to promote the conversion of Foxp3^−^ into Foxp3^+^ cells, thus contributing to tolerance (15), we asked whether hCG is able to support IL-10 production by B cells. Indeed, hCG blockage hampered the positive effect of trophoblasts on IL-10 production. The addition of recombinant hCG had the same effect as first trimester trophoblast supernatant and could boost the production of IL-10 by CD19^+^CD24^hi^CD27^+^ cells. Thus, hCG has an important role in switching B cells to a phenotype that supports pregnancy. Interestingly, other pregnancy hormones that are produced, albeit not exclusively, by the placenta (e.g., P4 and E2) had no effect on B cell phenotype, remarking the unique properties of hCG. Interestingly, hCG has been reported to have other immunomodulatory functions: it can modulate the phenotype of macrophages and DCs is able to induce uNK cell proliferation, suppress Th1 cytokine production by T cells and generate Treg out of naïve T cells [revised in Ref. (16)].

B lymphocytes are best known for their capability to secrete Ig, which enables them to identify and neutralize foreign antigens. Despite of an overall four-chain structure, antibodies differ considerably in their glycosylation sites, both in number and location (macroheterogeneity) and in glycan structures (microheterogeneity). Such variations within the conserved glycosylation site on IgG, for example, modulate its biological activity resulting in different effector functions. During normal pregnancy, IgG glycosylation changes showing an increase of galactosylation and sialylation and an accompanying decrease in the percentage of bisecting GlcNAc (19, 35, 36). Structural changes in glycosylation are one mechanism to favor pregnancy success by reducing alloreactive immune responses (20). Previous studies demonstrated that asymmetrically glycosylated IgG antibodies (AAbs) are IgG molecules with their Fab portion(s) containing a glycan chain; they are increased in maternal serum and placental tissue during normal human pregnancy, particularly in response to Th2 interleukins, namely IL-4, IL-6, and IL-10 (21). Moreover, hormonal influences and specifically P4 have been shown to provoke their release (22). This seems to be relevant for pregnancy outcome as women with recurrent spontaneous abortion presented lower asymmetric antibody (AAbs) levels than women undergoing normal pregnancies (23). These antibodies are likely to block immune responses, including complement fixation and phagocytosis, thus protecting the fetus from a potential immune attack by the mother.

We next studied the effect of hCG on antibody quantity and quality. Neither hCG nor the other tested hormones induced a change in the concentration of Igs produced after 12-day culture. Recently, much attention has been put on differentially glycosylated antibodies (24, 37, 38). While no changes in galacosylation, bisection, fucosylation, or sialyation of the Fc region could be observed after *in vitro* treatment of B cells with pregnancy hormones, we provide evidences that, at least *in vitro*, hCG increased the production of asymmetrically glycosylated IgG antibodies (AAbs). So-called AAbs are IgG molecules with their Fab portion(s) containing a glycan chain. These antibodies have been described some time ago and were described to be pregnancy protective (20). Indeed, they are not only present in the placenta from normal pregnant women (39) but also increased in the maternal serum during pregnancy compared to non-pregnant individuals (23). These antibodies have been postulated to bind detrimental antibodies in order to block immune responses, including complement fixation and phagocytosis, thus protecting the fetus from a potential immune attack by the mother. Patients with recurrent spontaneous abortion had low levels of these AAbs. It has been postulated that P4 is able to induce the asymmetric glycosylation (22). Gutiérrez and colleagues identified IL-6 as an important factor modulating AAbs (40), and it is known that trophoblast-derived IL-6 interacts with IL-6-R on the trophoblasts, resulting in hCG release (41). This is for sure a quite interesting pathway to study in the future. In contrast to the favorable effect proposed for AAbs on pregnancy outcome, so-called natural antibodies are suggested to provoke autoreactivity due to their polyreactive nature (42). Natural antibodies are produced by a subset of B cells called B1a B cells, whose number is significantly reduced during third trimester of healthy pregnant women, as our laboratory has shown (43). The B1a B cell count, however, remained elevated in preeclamptic patients and produce autoantibodies against angiotensin II type 1 receptor responsible for disease manifestation (43). Abnormally increased levels of hCG may be responsible for the autoantibody production (43). Thus, hCG emerges as a hormone with a relevant role in B cell regulation: at levels compatible with normal pregnancies, it supports the function of B cells that contribute to tolerance, while at levels observed in pathologic pregnancies, it can stimulate B1 a B cells to produce antibodies that are pregnancy deleterious [revised in Ref. (25)]. In this context, it is for sure interesting to study how pregnancy friendly vs. pregnancy dangering cytokines work together with hormones to modulate B cells.

Alpha-fetoprotein is an immunomodulatory glycoprotein, mainly produced by the fetal liver and able to diffuse across the placental barrier into the maternal blood circulation. A series of studies have proposed that AFP is able to regulate other immune cells. Tumor-derived AFP causes suppression of T lymphocyte responses (44). Here, we intended to understand the effect of fetus-derived AFP on B cells, in particular, how AFP impacts on their capacity to produce IL-10. We found that concentrations comparable to the levels observed in maternal serum during pregnancy had no effect on B cell phenotype and IL-10 production. However, when used at concentrations observed at the fetal side, AFP significantly induced total B cell apoptosis and death. Further experiments assessing the caspase activity supported this notion and demonstrated that the activity of caspase-3 and -7 was enhanced when total B cells were treated with elevated levels of AFP. Um and colleagues who illustrated some time ago that AFP induces apoptosis in antigen-presenting cells (APCs), particularly DCs. AFP-treated DCs also produced lower levels of IL-12 and TNF-α, thereby downscaling a Th1 response (18). The mechanisms underlying AFP-induced apoptosis are yet not known but it is known that at least in DCs leads them to become dysfunctional and change their cytokine secretion profile (18). Our data strongly suggest that AFP at concentrations present in fetal compartments drives B cells to death. This may constitute one unexplored mechanism of fetal protection. By driving B cells into apoptosis, AFP hinders them to reach fetal compartments where they could either act as APCs or initiate the production of detrimental Igs.

In conclusion, we have showed that a particular soluble factor produced by the placenta, namely hCG, is essential to modulate the phenotype of B cells and their ability to produce IL-10. Furthermore, hCG promotes the generation of pregnancy protective AAbs. Thus, the fetus governs its own tolerance by secreting hCG. AFP is able to drive B cell into apoptosis if used at fetal concentrations. This may also represent a protection mechanism induced by the fetus. Overall, our data impressively show how substances produced by the fetus itself are active participants of its own survival. Our findings contribute to the understanding of the fascinating phenomenon of pregnancy tolerance.

## Author Contributions

FF performed experiments, analyzed data, and greatly contributed to manuscript preparation. AC, NT, and AB performed experiments and analyzed data. IB-D, MW, and S-DC contributed to data analysis and interpretation. AS and ACZ designed and supervised the work, and analyzed data. ACZ provided funding and wrote the final version of the paper. All the authors approved the final version of the manuscript.

## Conflict of Interest Statement

The authors declare that the research was conducted in the absence of any commercial or financial relationships that could be construed as a potential conflict of interest. The reviewers CL and RR declared a shared affiliation, though no other collaboration, with two of the authors (AC and NT) to the handling editor, who ensured that the process nevertheless met the standards of a fair and objective review.

## Abbreviations

AAbs, asymmetric antibody; AFP, alpha-fetoprotein; APC, antigen-presenting cell; DC, dendritic cell; E2, estradiol; hCG, human chorionic gonadotropin; IL-10, interleukin 10; P4, progesterone.
