# Supplementary material for: Maternal and Fetal Mechanisms of B Cell Regulation during Pregnancy: Human Chorionic Gonadotropin Stimulates B Cells to Produce IL-10 While Alpha-Fetoprotein Drives Them into Apoptosis
Source: Front Immunol. 2016 Dec 8;7:495. doi: 10.3389/fimmu.2016.00495 (PMC5144100; doi:10.3389/fimmu.2016.00495)
Supplement: Supplementary file 1 [file Presentation_1.PDF]

Supplementary Material

Maternal and fetal mechanisms of B cell regulation during pregnancy: human Chorionic Gonadotropin stimulates B cells to produce IL-10 while alpha-fetoprotein drives them into apoptosis

Franziska Fettke[1,5], Anne Schumacher[1], Andrea Canellada[2], Natalia Toledo[2], Isabelle Bekeredjian-Ding[3], Albert Bondt[4], Manfred Wuhrer[4], Serban-Dan Costa[5], Ana Claudia Zenclussen[1*]

***Correspondence:**

Corresponding Author: Prof. Dr. Ana Claudia Zenclussen, Experimental Obstetrics and Gynecology, Medical Faculty, Otto-von-Guericke University Magdeburg, Gerhart-Hauptmann-Straße 35, 39108 Magdeburg, Germany. Tel.: +49 -391-67-17460, Fax +49-391-67-17440. E-mail: ana.zenclussen@med.ovgu.de

Supplementary Figure 1: CD19$^+$ B cell purity following magnetic isolation was above 95 %

B cells were isolated from PBMCs using the B cell isolation Kit, using MS/LS columns and MidiMACS Separator. Our flow cytometry analysis showed that CD19$^+$ (FITC) B cells had a purity above 95 %.

Supplementary Figure 2: Schematic diagram of 3 culture conditions

Schematic diagram of the three culture conditions for B cells and their main characteristics; adding recombinant pregnancy hormones and AFP concentrations for flow cytometry assessment and IL-10 quantification (A), Ig quantification (B) and co-culture of B cells and JEG-3 cells.

Supplementary Figure 3: IL-10 cytokine secretion was unaffected by pregnancy hormones

(A) CD19$^+$ B cells were magnetically isolated from human PBMCs. Stimulated B cells (CD40L/CpG) were co-cultured with JEG-3 trophoblast cells for 24 h in charcoaled medium in the presence or absence of an anti-hCG antibody. IL-10 secretion in supernatants was analyzed by ELISA. JEG-3 co-culture increased IL-10 secretion.

Supplementary Figure 4: Supernatant from JEG-3 cells stimulated IL-10 production by CD19$^+$ cells. hCG and CD40L/CPG had also a stimulatory effect on IL-10 production, while their combined addition the culture boosted IL-10 production

CD19$^+$ B cells were magnetically isolated from PBMCs from female donors (n=5) and cultured cultured either with JEG-3 supernatant, or in medium (DMEM containing 10% Fetal Bovine Serum, 1% Penicillin/Streptomycin) with hCG (100 mIU/ml), or stimulated with (CD40L/CpG, 1 μg/mL and

10 μg/mL respectively) or CD40L/CPG with hCG (same dose) for 48 hs. The addition of JEG-3 supernatant boosted the capacity of B cells to produce IL-10 as analyzed by floy cytometry. The addition of hCG or CD40L/CPG provoked the same effect, while the simultaneous Statistical analysis was carried out by repeated measures one-way ANOVA followed by Bonferroni correction for multiple analysis.

Supplementary Figure 5: IL-10 secretion after hormonal stimulation

Addition of recombinant hCG (100mIU/ml), P4 (30 ng/ml), E2 (1000 pg/ml) or the combination of P4 and E2 did not significantly alter IL-10 secretion by stimulated B cells. Unstimulated B cells served as controls. Statistical analysis was carried out by repeated measures one-way ANOVA followed by Bonferroni correction for multiple analysis.

Supplementary Figure 6: Total IgM, IgG and IgA were unaffected by pregnancy hormones and AFP

Quantification of human IgM, IgG and IgA assessed in supernatants after treatment with pregnancy hormones (**A**) or AFP (**B**) and incubation for 12 d was performed by ELISA. No significant differences for all Igs could be detected following treatment with hCG, P4, E2 and P4/E2 or treatment with AFP (**A** and **B**). Results represent means ± SEM. Statistical analysis was carried out by repeated measures one-way ANOVA followed by Bonferroni correction for multiple analysis. *p ≤ 0,05.

Supplementary Figure 7: IgG glycosylation at their Fc region was not affected by pregnancy hormones or AFP treatment

B cells were cultured for 12 d in charcoaled medium (control) or charcoaled medium with stimulation (CD40L/CpG) in the presence of recombinant hCG (100 mIU/ml), P4 (30 ng /ml), E2 (1000 pg/ml)

or the combination of P4 and E2 (**A**) or with AFP at a concentration found in maternal serum (1st trimester: 0.015 µg/ml, 2nd trimester: 0.06 µg/ml, 3rd trimester: 0.2 µg/ml), or AFP at a concentration found in fetal serum (50 µg/ml) (**B**). Glycopeptide analysis did not reveal statistical differences with regards to IgG1 galactosylation, bisection, fucosylation, or sialylation, and neither for combined IgG2 and 3 glycosylation traits (data not shown).

Supplementary Figure 8: Dot plots depicting Annexin V/PI positive CD19$^+$ cells after stimulation with CD40L/CPG and CD40L/CPG + hCG.

Isolated CD19$^+$ B cells were stimulated by CD40L/CpG, stimulated and exposed to 50 µg/ml AFP or incubated in standard medium as a control for 24 h. The percentage of viable, apoptotic and necrotic cells in the total B cell population was assessed using the FITC Annexin V Apoptosis Detection kit with flow-cytometric analysis. Dot plots indicate representative dot plots for early apoptotic Annexin$^+$ cells (right bottom panel) or late apoptotic/necrotic Annexin$^+$/PI$^+$ cells (right upper panel).

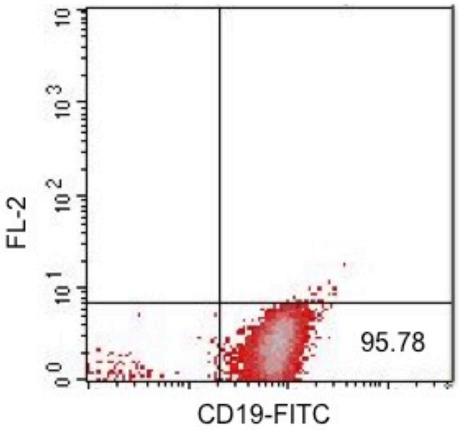

Supplementary Figure 1

(A)

(B)

(C)

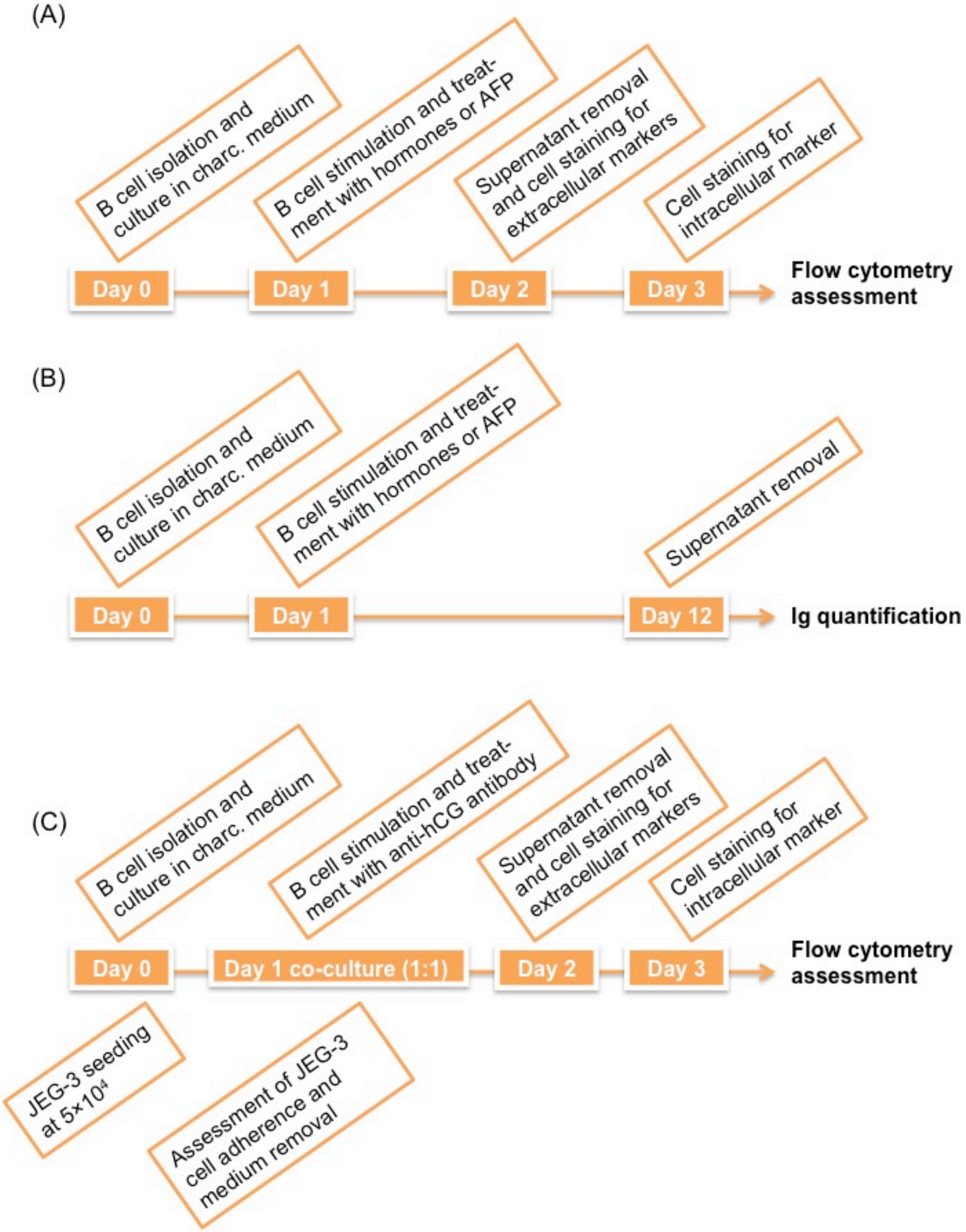

Supplementary Figure 2

(A)

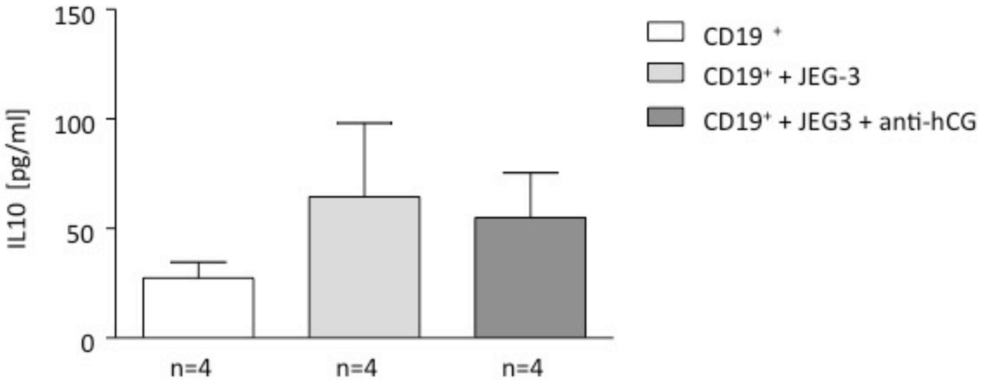

Supplementary Figure 3

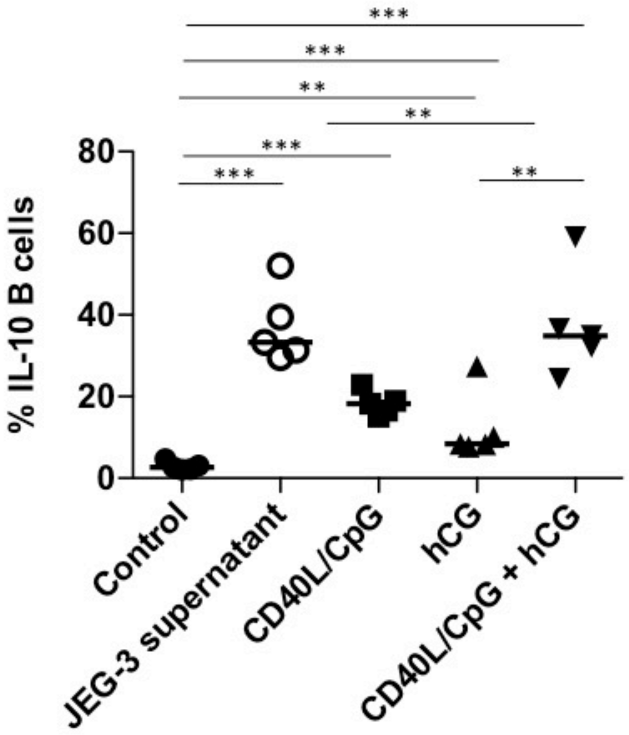

Supplementary Figure 4

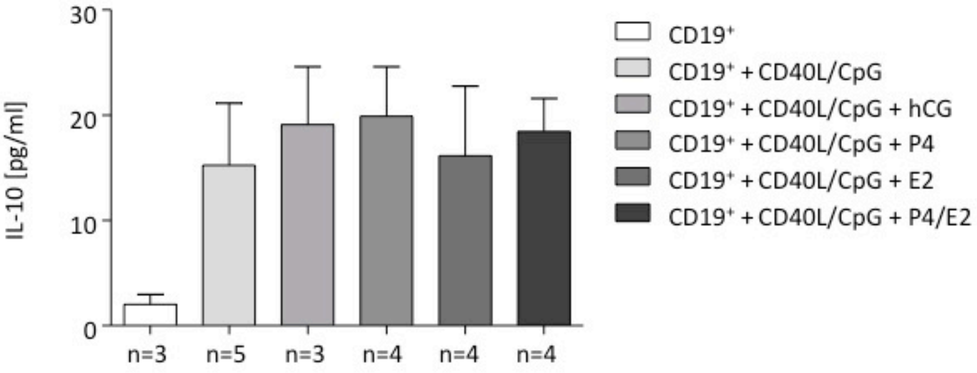

Supplementary Figure 5

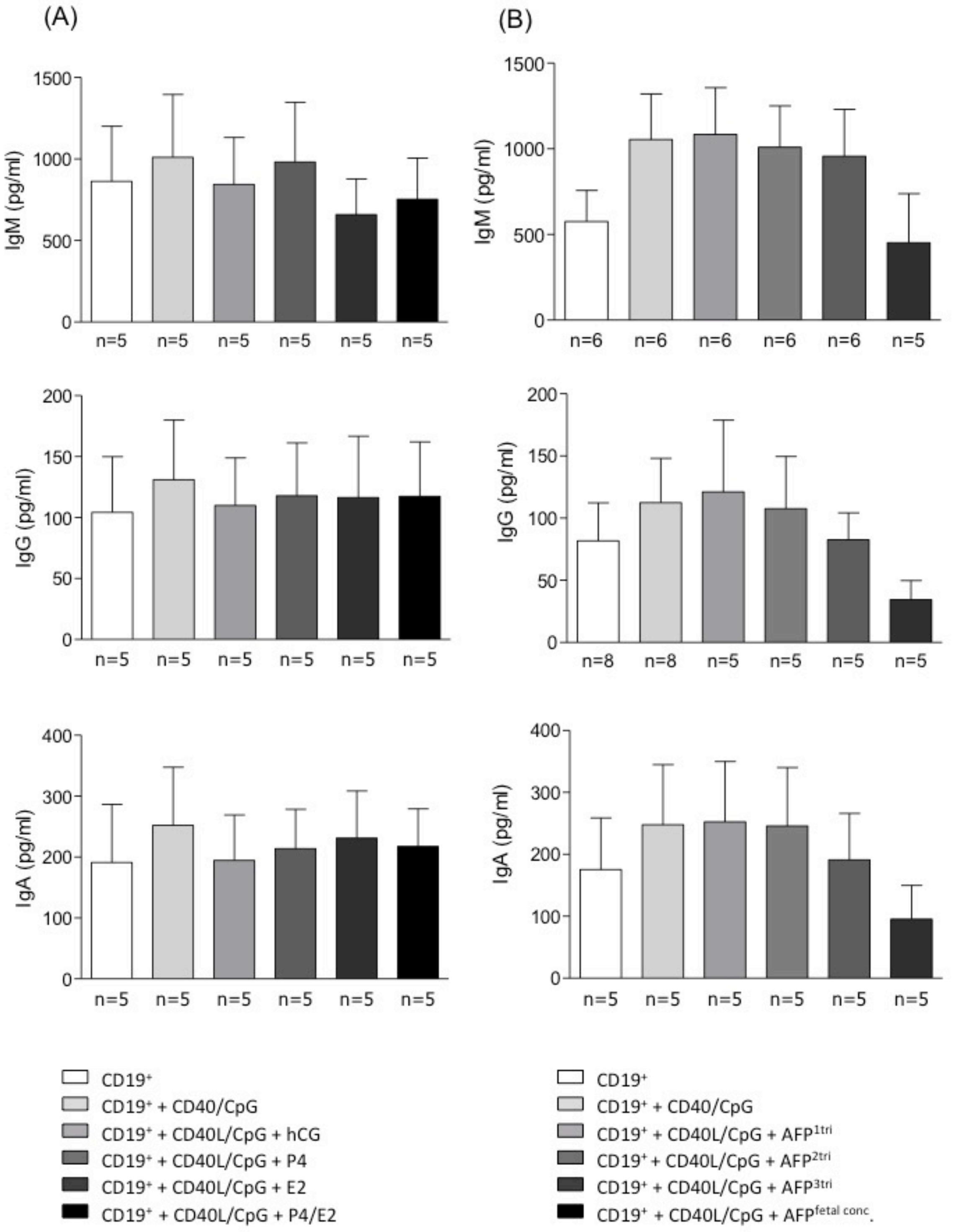

Supplementary Figure 6

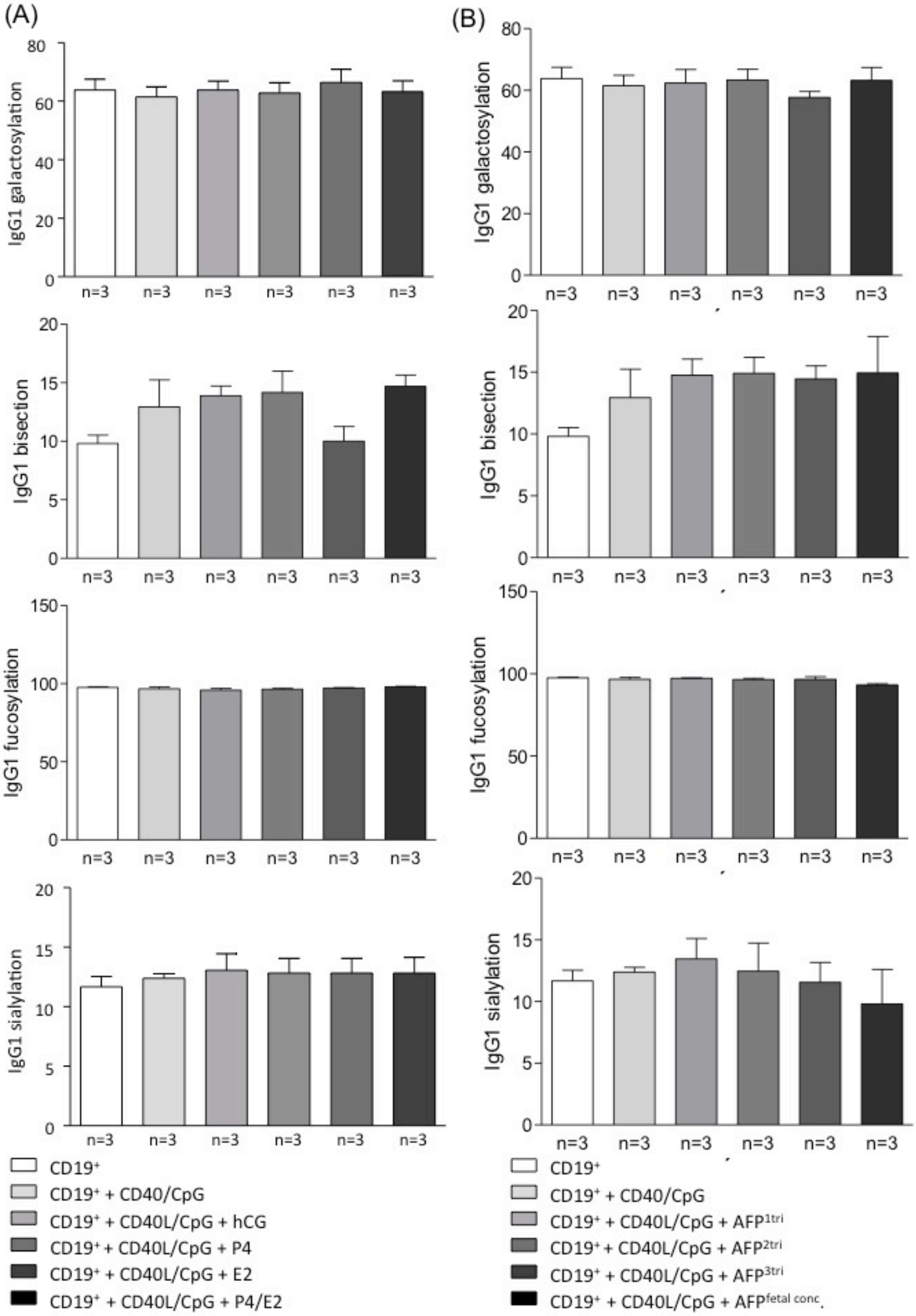

Supplementary Figure 7

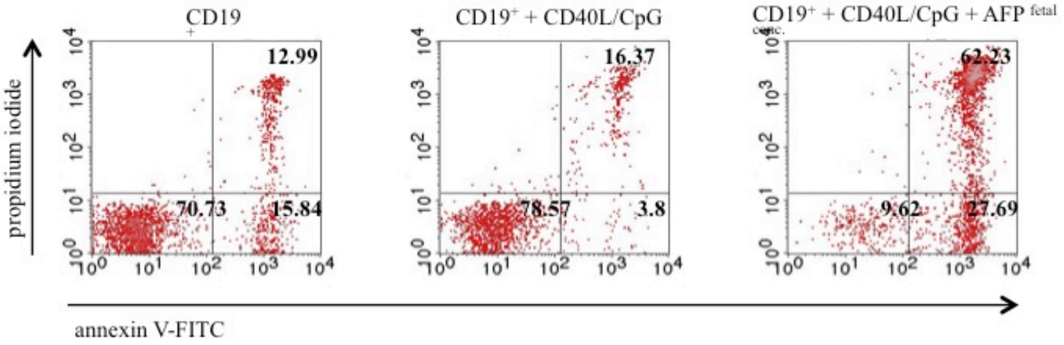

Supplementary Figure 8
